# Temporal requirements of insulin/IGF-1 signaling for proteotoxicity protection

**DOI:** 10.1111/j.1474-9726.2009.00541.x

**Published:** 2010-04

**Authors:** Ehud Cohen, Deguo Du, Derek Joyce, Erik A Kapernick, Yuli Volovik, Jeffery W Kelly, Andrew Dillin

**Affiliations:** 1 Howard Hughes Medical Institute, Glenn Center for Aging Research, Molecular and Cell Biology Laboratory, The Salk Institute for Biological Studies10010 North Torrey Pines Road, La Jolla, CA 92037, USA; 2 The Institute for Medical Research Israel – Canada, the Hebrew University of Jerusalem Medical SchoolEin-Kerem, Jerusalem 91120, Israel; 3 Departments of Chemistry and Molecular and Experimental Medicine and The Skaggs Institute of Chemical BiologyThe Scripps Research Institute, 10550 North Torrey Pines Road, La Jolla, CA 92037, USA

**Keywords:** *Caenorhabditis elegans*, insulin/IGF-1 signaling, longevity, proteotoxicity

## Abstract

Toxic protein aggregation (proteotoxicity) is a unifying feature in the development of late-onset human neurodegenerative disorders. Reduction of insulin/IGF-1 signaling (IIS), a prominent lifespan, developmental and reproductive regulatory pathway, protects worms from proteotoxicity associated with the aggregation of the Alzheimer’s disease-linked Aβ peptide. We utilized transgenic nematodes that express human Aβ and found that late life IIS reduction efficiently protects from Aβ toxicity without affecting development, reproduction or lifespan. To alleviate proteotoxic stress in the animal, the IIS requires heat shock factor (HSF)-1 to modulate a protein disaggregase, while DAF-16 regulates a presumptive active aggregase, raising the question of how these opposing activities could be co-regulated. One possibility is that HSF-1 and DAF-16 have distinct temporal requirements for protection from proteotoxicity. Using a conditional RNAi approach, we found an early requirement for HSF-1 that is distinct from the adult functions of DAF-16 for protection from proteotoxicity. Our data also indicate that late life IIS reduction can protect from proteotoxicity when it can no longer promote longevity, strengthening the prospect that IIS reduction might be a promising strategy for the treatment of neurodegenerative disorders caused by proteotoxicity.

## Introduction

Aging is the major risk factor for the development of late-onset human neurodegenerative disorders, including Huntington’s disease (HD) and Alzheimer’s disease (AD) (Amaducci & Tesco, 1994), both linked to aberrant protein aggregation (Selkoe, 2003). HD is associated with the aggregation of expanded poly-glutamine stretches (polyQ) in the protein huntingtin (Bates, 2003), while AD is associated with the aggregation of the Aβ peptide. Although it is not entirely clear why these disorders emerge late in life, it is plausible that the aging process plays an active role in enabling their onset. One theory suggests that biological activities that defend against toxic protein aggregation (proteotoxicity) decline with age (Cohen *et al.*, 2006). The insulin/IGF-1 signaling (IIS) pathway is a prominent aging regulator and lifespan determinant in worms (Kenyon *et al.*, 1993; Kenyon, 2001), flies (Giannakou & Partridge, 2007) and mice (Bluher *et al.*, 2003; Holzenberger *et al.*, 2003; Taguchi *et al.*, 2007). Reduced IGF signaling was recently shown to be linked to the regulation of human lifespan (Suh *et al.*, 2008; Willcox *et al.*, 2008; Flachsbart *et al.*, 2009), suggesting that the longevity functions of this pathway are conserved from worms to humans. In the nematode *Caenorhabditis elegans*, the sole insulin/IGF-1 receptor, DAF-2, mediates the phosphorylation, via downstream kinases, of the forkhead-like transcription factor, DAF-16, prevents it from entering the nucleus and compromises DAF-16 target gene expression (Lee *et al.*, 2001). This results in a shortened lifespan and elevated stress sensitivity. Thus, genetic knockdown of *daf-2* enables DAF-16 to enter the nucleus and creates long-lived, stress-resistant worms (Kenyon, 2005). DAF-16 is critically required for reduced IIS to mediate longevity in worms, as *daf-16* knockdown by RNAi or mutation abolishes the increased longevity of *daf-2* mutant animals (Kenyon *et al.*, 1993; Tissenbaum & Ruvkun, 1998; Lee *et al.*, 2001).

The heat shock factor 1 (HSF-1) is also essential for lifespan extension facilitated by reduced IIS (Hsu *et al.*, 2003; Morley & Morimoto, 2004). HSF-1 is predominantly regulated by trimer formation and nuclear entry upon heat shock induction (Sarge *et al.*, 1993). DAF-16 and HSF-1 have shared and distinct regulation of downstream genes, especially those of the chaperone class. However, hitherto it is unknown whether *hsf-1* activity is directly regulated by the IIS pathway.

Reduced IIS protects worms from various stress conditions, including thermal (Lithgow *et al.*, 1995) and oxidative stress (Honda & Honda, 1999). Recent studies indicate that an IIS reduction can also protect worms from polyQ (Morley *et al.*, 2002; Hsu *et al.*, 2003) and Aβ (Cohen *et al.*, 2006) aggregation associated proteotoxicity. These results indicate that the IIS coordinately regulates the aging process and protein homeostasis, suggesting that activities that defend against toxic protein aggregation decline with age (Cohen & Dillin, 2008).

To characterize the detailed mechanistic links between the IIS and toxic protein aggregation, we utilized transgenic worms that express the human Aβ_1–42_ minigene driven by a muscle specific promoter (Aβ worms) (Link, 1995), resulting in muscular dysfunction and age-dependent progressive paralysis of the worm population. We reported that IIS reduction protected animals from Aβ_1–42_ mediated paralysis in a DAF-16 and HSF-1 dependent manner when administered during development and adulthood [*daf-2*, *daf-16* and *hsf-1* RNAi treatments affects neither Aβ expression levels nor the Aβ total protein amounts (Cohen *et al.*, 2006)]. HSF-1 and DAF-16 regulate opposing protective activities; HSF-1 influences disaggregation while DAF-16 mediates the formation of larger, plausibly less toxic aggregates (Cohen *et al.*, 2006). Our findings suggest that an aging associated decline in these IIS regulated protective activities enables proteotoxicity to manifest late in life (Cohen & Dillin, 2008) and point to IIS reduction as a promising approach to develop neurodegenerative therapies (Morimoto, 2006). Recently, we have extended our studies to mammals and found that aging alteration by reducing IGF signaling protects mice from behavioral, pathological and biochemical phenotypes associated with AD-like disease (Cohen *et al*., 2009).

However, the IIS pathway controls multiple processes, including development, reproduction and longevity. The complexity of the physiological processes regulated by this pathway poses a significant hurdle utilizing and validating it as a bona fide target for neurodegenerative disease therapies. Most importantly, hitherto it is unknown whether IIS reduction late in life, the stage at which most human neurodegenerative disorders onset, could provide protection from proteotoxicity. To address this issue, we asked whether it was possible to separate the physiological requirements of the IIS pathway for protection against proteotoxicity from its developmental, reproductive and most importantly, its requirement for the determination of lifespan.

## Results

### IIS can regulate proteotoxicity independent of longevity

**Fig 1:**
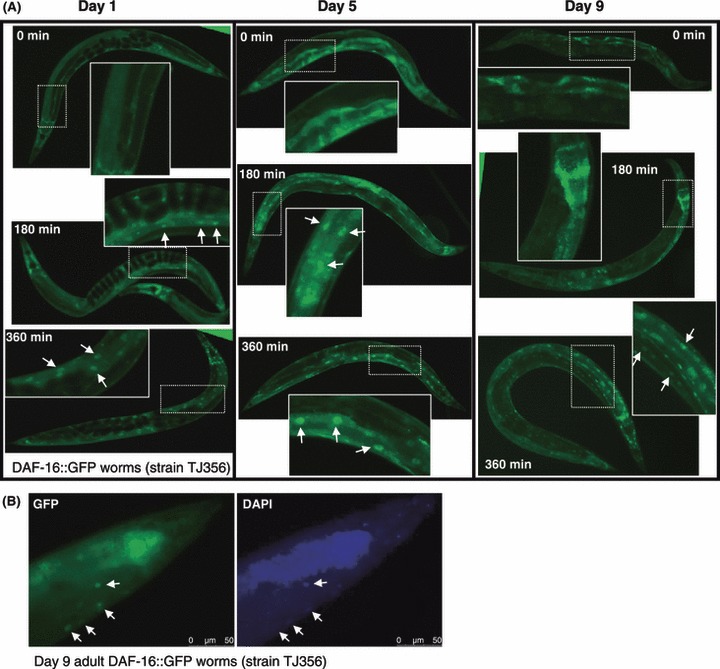
Late life insulin/IGF-1 signaling reduction promotes DAF-16 nuclear localization. (A) DAF-16::GFP expressing worms (strain TJ356) were grown on control bacteria (EV) to either day 1 or 9 of adulthood, and transferred onto *daf-2* RNAi bacteria. Green fluorescent protein (GFP) signal was visualized 0, 3 or 6 h after the transfer. Six hours after transfer, GFP signal in worms that were treated during early and late adulthood were concentrated in the nuclei. (B) Day 9 DAF-16::GFP worms were placed on *daf-2* RNAi for 6 h, fixed and stained with DAPI. Co-localization of the DAPI and GFP signals (arrows) confirmed the nuclear localization of DAF-16 in day 9 old worms that were fed *daf*-2 RNAi.

We have addressed this question using Aβ and DAF-16 reporter worm strains taking advantage of our understanding of the IIS pathway and its role in the highly conserved aging program of *C. elegans.* First, we tested whether the application of *daf-2* RNAi late in life affects the intra-cellular localization of DAF-16, as it does early in life, using worms expressing green fluorescent protein (GFP) fused to functional DAF-16 [strain TJ356 (Henderson & Johnson, 2001)]. In worms at days 1, 5 and 9 of adulthood, DAF-16 efficiently enters the nucleus within 6 h after transferring the worms onto *daf-2* RNAi expressing bacteria [Fig. 1A,B, *daf-2* and *daf-16* RNAi treatments effectively reduced target gene expression (Dillin *et al.*, 2002)]. These observations indicate that *daf-2* reduction by RNAi similarly affects DAF-16 localization whether applied in early adulthood (day 1) or late in adulthood (day 9) when it can no longer extend lifespan (Dillin *et al.*, 2002).

**Fig 2:**
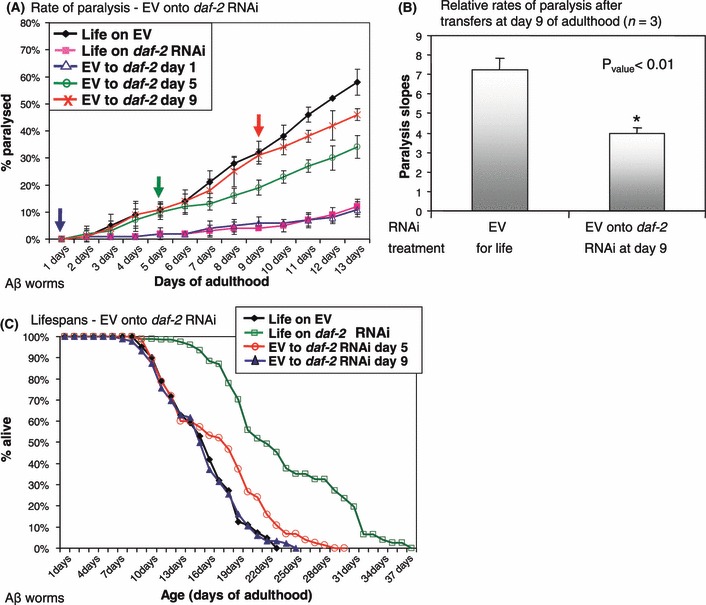
Timing requirements for *daf-2* RNAi mediated protection from Aβ proteotoxicity. (A) Aβ worms were transferred from empty vector (EV) bacteria onto *daf-2* RNAi bacteria at either day 1, 5 or 9 of adulthood. Paralysis rates decreased upon transfer to *daf-2* RNAi compared to EV-grown control worms at all tested ages. (B) Three independent repeats of (A) indicate that the reduction of Aβ toxicity observed in worms transferred at day 9 is reproducible and significant. (C) Lifespan of control Aβ worms grown throughout life on EV bacteria and their counterparts which were transferred from EV onto *daf-2* RNAi bacteria at day 9 of adulthood are undistinguishable (mean lifespan: 15.48 and 15.35 days respectively, *P*_value_ = 0.772). Lifespan of worms that were transferred from EV bacteria onto *daf-2* RNAi at day 5 of adulthood were significantly shorter than these of their counterparts which were grown on *daf-2* RNAi throughout life (mean lifespan: 17.04 and 23.74 respectively, *P*_value_ = 3.55E-09).

To test whether late life IIS reduction and the subsequent DAF-16 relocalization into the nucleus, could protect from age onset proteotoxicity associated with human Aβ_1–42_ peptide expression, we utilized the Aβ worm model. To temporally attenuate IIS, Aβ worms were hatched and developed on control bacteria harboring an empty vector (EV) and then transferred onto *daf-2* RNAi bacteria at either day 1, 5 or 9 of adulthood (Fig. 2A, Fig. S1). *daf-2* RNAi protected worms from paralysis associated with Aβ proteotoxicity when applied during early adulthood, days 1 and 5 of adulthood, the time window in which it can promote longevity (Dillin *et al.*, 2002). Interestingly, application of *daf-2* RNAi during late adulthood, day 9, also suppressed further Aβ proteotoxicity within the worm population (Fig. 2A,B). Importantly, this late life protective effect was observed even if *daf-2* RNAi was applied relatively late in life beyond the time window in which it could extend lifespan (Fig. 2C blue, Supporting Fig. S2 and (Dillin *et al.*, 2002). We also tested the effect on lifespan of transferring Aβ worms from EV bacteria onto *daf-2* RNAi bacteria at day 5 of adulthood and found that the lifespan extension was relatively small (Fig. 2C red). This observation is consistent with the results published for wild-type worms transferred at the same time (Dillin *et al.*, 2002). Thus, although the longevity and anti-proteotoxicity functions of reduced IIS overlap in early adulthood (days 1–5), they can be temporally dissociated late in life (day 9 of adulthood).

### Late life IIS reduction promotes aggregation of Aβ

Previously, we found that protection from Aβ proteotoxicity by reduced IIS is associated with the accumulation of high molecular weight (high-MW) Aβ aggregates (Cohen *et al.*, 2006). To examine whether a similar mechanism underlies the late life protection mediated by *daf-2* RNAi treatment, we adopted a biochemical approach to measure the content of high-MW Aβ aggregates in worms that were treated either early or late in life with *daf-2* RNAi.

**Fig 3:**
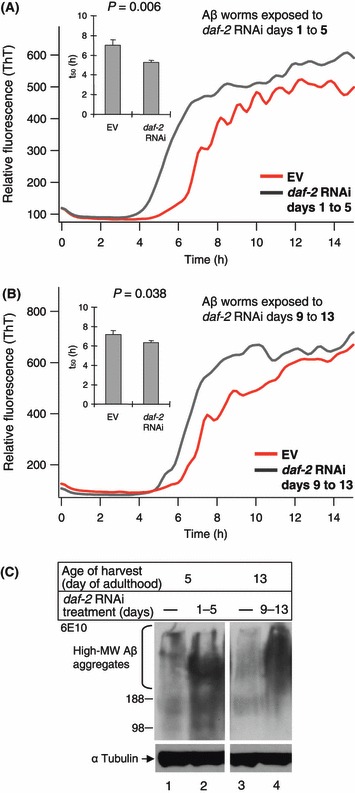
(A, B) *In-vitro* kinetic aggregation assay reveals that Aβ worm homogenates that were treated with *daf-2* RNAi either early (days 1–5 of adulthood) (A) or late (days 9–13 of adulthood) (B) in life had higher Aβ seed content compared to their control untreated age-matched counterparts. (C) Western blot analysis using Aβ monoclonal antibody (6E10) indicated that high-MW Aβ aggregates contents in insoluble fractions (debris) of Aβ worm that were treated with *daf-2* RNAi either early (lane 2) or late (lane 4) in life were higher compared to their control untreated age-matched counterparts (lanes 1 and 3 respectively).

Uniform length Aβ aggregates derived from the sonication of homogenized Aβ worms hasten an *in-vitro* Aβ_1–40_ polymerization reaction in a dose-dependent fashion (Hasegawa *et al.*, 1999; Cohen *et al.*, 2006). Therefore, the higher content of fibrillar Aβ aggregates in the worms shorten the lag phase associated with the initiation of Aβ aggregation in the test tube owing to the process of seeding or bypassing the requirement for nucleation. The dye thioflavin-T (ThT) selectively binds to Aβ fibrils, shifting the wave length of its fluorescent emission and enabling accurate measurement of the *in-vitro* Aβ fibrilization reaction. The *in-vitro* kinetic aggregation assay was utilized to measure the content of fibrillar Aβ aggregates within Aβ worms (Cohen *et al.*, 2006). Aβ worms were hatched on control bacteria (EV) and transferred onto *daf-2* RNAi bacteria at either early age (days 1–5 of adulthood) or late in life (days 9–13 of adulthood). In all cases, worms were cultured on the *daf-2* RNAi bacteria for identical amounts of time, 4 days, prior to harvest for biochemical analysis. The worms were homogenized and separated by low-speed centrifugation (845 *g*, 5 min desktop centrifuge) into soluble [post debris supernatant (PDS)] and insoluble (debris) fractions. To provide robust quantification results independent from the initial distribution of fibril lengths, the PDS was sonicated for 10 min to break worm derived Aβ fibrils into unified length. Using the *in-vitro* kinetic aggregation assay, we compared the seeding efficiency of sonicated worm PDS fractions from animals treated with *daf-2* RNAi early or late in life. Worms treated with *daf-2* RNAi, whether early in life (days 1–5, Fig. 3A), or later in life (days 9–13, Fig. 3B) exhibited an increased amount of Aβ fibrils as reflected by the increased seeding efficiency of their sonicated PDS compared to untreated age-matched counterparts.

In addition to the *in-vitro* kinetic assay, we also measured the high-MW Aβ aggregates in the insoluble fractions of the early and late-life treated worm groups using western blot analysis and an Aβ antibody (clone 6E10). We found that both early and late life *daf-2* RNAi treatments afforded increased amounts of high-MW Aβ aggregates (Fig. 3C, lanes 2 and 4 respectively) relative to age-matched untreated controls (Fig. 3C, lanes 1 and 3).

Taken together, attenuation of *daf-2* either early or late in life results in nuclear localization of DAF-16, protection from further Aβ associated proteotoxcity and increased amounts of high molecular weight Aβ aggregates. These observations suggest that IIS reduction either early or late in life, can protect from age onset proteotoxicity by invoking a mechanism that converts toxic aggregates into larger, less toxic high molecular weight aggregates.

### DAF-16 is required during adulthood to protect from Aβ proteotoxicity

**Fig 4:**
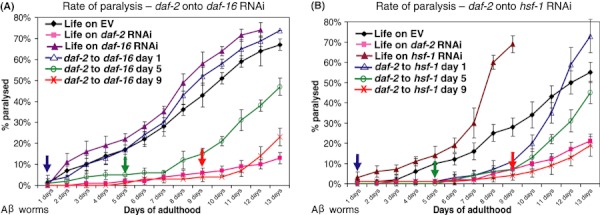
Timing requirements for *daf-16* and *hsf-1* RNAi mediated protection from Aβ proteotoxicity (A) Aβ worms were grown on *daf-2* RNAi bacteria throughout life or were transferred to *daf-16* RNAi bacteria on either day 1, 5 or 9 of adulthood. Development on *daf-2* RNAi did not protect the worms from Aβ mediated paralysis compared to EV and *daf-16* RNAi controls. Worm transferred from *daf-2* onto *daf-16* RNAi at either day 5 or 9 of adulthood were protected for 2 days after exposure to *daf-16* RNAi. (B) Aβ worms developed on *daf-2* RNAi were transferred onto *hsf-1* RNAi at either day 1, 5 or 9 of adulthood. Development on *daf-2* RNAi protected the worms from paralysis for 8 days while animals transferred at day 5 were protected for one additional day. All panels display one of three independent experiments.

DAF-16 is essential for the counter-proteotoxic activity of reduced IIS (Morley *et al.*, 2002; Hsu *et al.*, 2003; Cohen *et al.*, 2006). Thus, we asked whether *daf-16* was required during the same time window as *daf-2* RNAi (reduced IIS) to protect animals from age onset proteotoxicity. We tested whether *daf-16* RNAi could directly eradicate the protection provided by IIS reduction by following the paralysis phenotype associated with expression of Aβ (Cohen *et al.*, 2006). Aβ worms that were hatched and developed on *daf-2* RNAi expressing bacteria transferred away from *daf-2* RNAi onto *daf-16* RNAi bacteria at either day 1, 5 or 9 of adulthood (*daf-16* RNAi reduces mRNA levels within 3 h, Supporting Fig. S4A) and the rates of paralysis within the worm populations were recorded (Fig. 4A). Analogous to its role in lifespan determination (Dillin *et al.*, 2002), *daf-2* reduction during development did not protect from proteotoxicity, as worms transferred from *daf-2* RNAi onto *daf-16* RNAi at day 1 of adulthood paralyzed at similar rates as control animals [removal of the worms from *daf-2* RNAi onto control bacteria did not abolish the counter-proteotoxic protective effect of *daf-2* RNAi through day 13 of adulthood, most likely due to the stability of the RNAi (Supporting Fig. S3)]. Equivalent to its role in lifespan determination, the developmental functions of *daf-2* (embryogenesis, larval development and reproductive timing) could be temporally separated from the anti-proteotoxicity function of reduced IIS. Therefore, *daf-16* is required to protect from Aβ_1–42_ toxicity only during adulthood.

Animals developed on *daf-2* RNAi and transferred onto *daf-16* RNAi at days 5 or 9 were temporarily protected, but eventually succumbed to proteotoxicity 2 days after the transfer (Fig. 4A). The 2-day phenotypic lag period might stem from the turnover of DAF-16 or protective proteins encoded by DAF-16 regulated genes. Alternatively, Aβ toxicity may take up to 2 days for full induction of paralysis in the worm model. Taken together, *daf-2* and *daf-16* have overlapping temporal requirements for protection from proteotoxicity that extend well into adulthood after the appearance of proteotoxic stress on the population.

### HSF-1 is predominantly required during larval development for proteotoxicity protection

Heat shock factor-1 is also critical for the anti-proteotoxicity activity of reduced IIS (Hsu *et al.*, 2003; Cohen *et al.*, 2006). To determine the timing requirements for *hsf-1,* Aβ worms were grown on *daf-2* RNAi and transferred onto *hsf-1* RNAi bacteria at either day 1, 5 or 9 of adulthood (Fig. 4B). Surprisingly, and in stark contrast to the *daf-16* RNAi experiments, worms transferred away from *daf-2* RNAi onto *hsf-1* RNAi at day 1 of adulthood did not exhibit paralysis until day 9 of adulthood, whereas animals developed and maintained on *hsf-1* RNAi throughout life readily succumbed to proteotoxicity. In addition, animals transferred to *hsf-1* RNAi at day 5 of adulthood were protected for only one additional day compared to their day 1 transferred counterparts. Animals transferred at day 9 showed no additional paralysis until termination of the experiment. Using quantitative PCR, we found that *hsf-1* RNAi readily reduced *hsf-1* gene expression 6 h after transferring the worms onto *hsf-1* RNAi bacteria (Supporting Fig. S4B), similar to *daf-2* and *daf-16* RNAi shown previously (Dillin *et al.*, 2002). *hsf-1* RNAi treatment has similar effects on the expression of its target gene HSP-16.2 (Link *et al.*, 1999) when applied early and late in life (Supporting Fig. S5). Thus, our observations indicate that unlike *daf-16*, *hsf-1* is required foremost during larval development, yet it is also needed for a lesser extent during adulthood to counter Aβ proteotoxicity.

## Discussion

**Fig 5:**
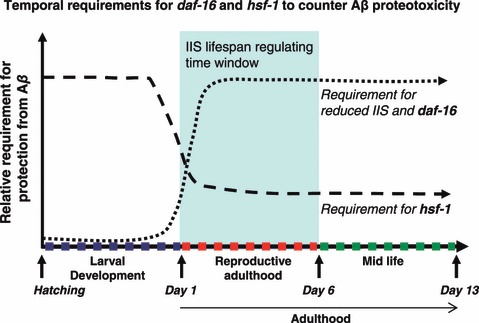
Timing requirement for reduced insulin/IGF-1 signaling (IIS), *daf-16* and *hsf-1* to counter Aβ proteotoxicity in the worm. IIS reduction during development has no effect on Aβ proteotoxicity if *daf-16* is attenuated at day 1 of adulthood. In contrast, IIS attenuation during reproductive adulthood and midlife protect from Aβ. This protection is associated with Aβ hyper-aggregation and dependent in *daf-16*. *hsf-1* is foremost required for protection from Aβ proteotoxicity during larval development but is also required for a lesser extent during early adulthood and midlife.

Collectively, our findings indicate that *daf-2, daf-16* and *hsf-1* are required up to advanced age for protection against muscular Aβ proteotoxicity and that late life IIS attenuation can protect from further damage without extending lifespan (Fig. 5). Furthermore, the developmental and reproductive functions of *daf-2* and *daf-16* could be separated from the proteotoxicity function of the IIS, which acts during adulthood.

It was surprising to find that *hsf-1* was predominantly required during development and again, to a lesser extent, during adulthood. The two-step requirement of *hsf-1* suggests that there might be an initiation phase required by *hsf-1* during development that is later acted upon during adulthood. Consistent with this idea, the histone deactylase SIRT1 is required to attenuate the heat shock response by directly deactylating HSF-1, enabling maintenance of HSF-1 for binding to its target genes (Westerheide *et al.*, 2009). Furthermore, SIRT1 expression declines with age in accordance with the age-dependent decline of the heat shock response. In future, it will be imperative to determine the temporal requirements of SIRT1 for longevity and proteotoxic stress, especially given the fact that SIRT1 also regulates the activity of FOXO in addition to HSF-1 (Brunet *et al.*, 2004).

The counter proteotoxic functions of the IIS are conserved from worms to mammals [Cohen *et al.* (in press)], suggesting that drugs directed to reduce IIS pathway, or its target genes, could delay neurodegeneration even if administered after diagnosis late in life, thereby possibly circumventing many of the potential deleterious effects of IIS reduction during development. The finding that *hsf-1* can function early, as well as later in life, to protect animals towards proteotoxicity provides a unique opportunity to study proteotoxic diseases during development, such as the aggressive forms of Huntington’s, Spinocerebral Ataxic diseases and juvenile Parkinsonism (Giasson & Lee, 2001).

## Experimental procedures

### Worm and RNAi strains

CL2006 (Link, 1995), CL2070, TJ356 and N2 worm strains were obtained from the Caenorhabditis Genetics Center (Minneapolis, MN). The worms were grown at 20 °C. To reduce gene expression, we used previously described (Dillin *et al.*, 2002) bacterial strains expressing dsRNA: EV (pAD12), *daf-2* (pAD48), *daf-16* (pAD43). *hsf-1* dsRNA expressing bacterial strain was from genomic RNAi library (J. Ahringer). Each RNAi bacterial colony was grown at 37 °C in LB with 100 μg mL^−1^ carbenicillin, and then seeded onto NG-carbenicillin plates supplemented with 100 mm Isopropyl β-D-1-thiogalactopyranoside (IPTG).

### DAF-16 localization assay

Synchronized TJ356 worms were grown on the EV control bacteria. At the indicated ages (days 1 or 9 of adulthood), 25 worms were transferred onto *daf-2* RNAi bacteria for the indicated time (0, 3 or 6 h). The worms were washed twice with M9, snap froze in liquid nitrogen and nuclei were labeled for 30 min using 4′,6-diamidino-2-phenylindole (DAPI) [200 ng mL^−1^; (Molecular Probes), Invitrogen, Carlsbad, CA, USA]. DAPI and GFP signals were visualized using a fluorescent microscope (Leica DM6000 B; Leica, Wetzlar, Germany).

### Paralysis assay

Synchronous CL2006 worm populations were grown on (NG) plates containing 100 μg mL^−1^ carbenicillin, spotted with *E. coli* cultures expressing dsRNA as indicated. On the first day of adulthood, 100 worms were placed on ten plates (ten animals per plate). The plates were divided randomly to five sets (two plates, 20 worms per set). The worms were tested every day for paralysis by tapping their noses with a platinum wire. Worms that moved their noses but failed to move their bodies were scored as ‘paralyzed’ and removed from the plates. To avoid scoring of old animals as paralyzed, paralysis assay terminated at day 13 of adulthood.

### RNA isolation and quantitative RT-PCR

Total RNA was isolated from synchronized populations of approximately 12 000 sterile worms (strain CF512) grown at 20 °C for each time point. Total RNA was extracted using QIAzol reagent (Cat #79306; QIAGEN, Hilden, Germany) and purified using RNeasy kit (QIAGEN #74104). cDNA was created using QuantiTect Probe RT-PCR Kit (QIAGEN #204443). For quantitative PCR reactions, dilutions of 1:10 were used. SybrGreen real-time qPCR experiments were performed as described in the manual using ABI Prism7900HT (Applied Biosystems, Foster city, CA, USA). Quantification was completed using SDS2.1 software (Applied Biosystems), normalizing to control levels of *act-1* cDNA.

1. *hsf-1* primer set 1: forward: TTGACGACGACAAGCTTCCAGT; reverse: AAAGCTTGCACCAGAATCATCCC.
2. *hsf-1* primer set 2: forward: GTCTCTGTCATGCAGCCAGG; reverse: TTGGGTCCGGCAGTTCC.
3. *daf-16* primers: forward: CTTCAAGCCAATGCCACTACC reverse: GGAGATGAGTTGGATGTTGATAGC.
4. *act-1* primer set: forward: GAGCACGGTATCGTCACCAA; reverse: TGTGATGCCAGATCTTCTCCAT.

### Lifespan analysis

Synchronized worm eggs were placed on master NG-carbenicillin plates seeded with the indicated RNAi bacterial strain and supplemented with 100 mm IPTG. The eggs were incubated at 20 °C until the worms reached L4 larval stage and were transferred onto small NG-carbenicillin plates (ten animals per plate). The worms were transferred onto freshly seeded plates every 4 days. Dead worms were scored daily. Lifespan analyses were conducted at 20 °C.

### *In-vitro* kinetic aggregation assay

Aβ worms were grown on RNAi bacterial strains as indicated. At the desired ages, the worms were washed twice with M9 and once more with phosphate-buffered saline (PBS) (RT), resuspended in 300 μL ice-cold PBS, transferred to a 2-mL tissue grinder (885482; Kontes, Vineland, NJ, USA) and homogenized. Crude homogenates were spun in a desktop microfuge (845 *g*, 3 min). Supernatants were transferred to new tubes and total protein concentrations were measured with BCA kit (Pierce, Rockford, IL, USA). Aβ_1–40_ peptide was diluted to a final concentration of 10 μm in phosphate buffer (150 mm NaCl, 50 mm Na-phosphate, pH 7.4) containing ThT (20 μm). PDS were sonicated for 20 min in a water bath sonicator (Model FS60; Fisher Scientific, Pittsburg, PA, USA). Proteinase K was added to final concentration of 200 ng mL^−1^, incubated for 2 h and supplemented with complete (EDTA free) protease inhibitors cocktail (Cat#1836170; Roche, Basel, Switzerland). The treated PDS solution was added to the assay at a final total protein concentration of 10 μg mL^−1^. Three aliquots (100 μL) of these solutions were transferred into wells of a 96-well microplate (Costar black, clear bottom) for each reaction. The plate was sealed and loaded into a Gemini SpectraMax EM fluorescence plate reader (Molecular Devices, Sunnyvale, CA, USA), where it was incubated at 37 °C. The fluorescence (excitation at 440 nm, emission at 485 nm) was measured from the bottom of the plate at 10 min intervals, with 5 s of shaking before each reading.

### Aβ blotting and detection

Equal numbers of Aβ worms were grown on either EV or *daf-2* RNAi bacteria as indicated. The worms were washed and homogenized as prepared for the *in-vitro* kinetic aggregation assay (see above). Total protein amounts were equalized using BCA kit as described above. Worm debris (insoluble fractions) were supplemented with 120 μL PBS, 40 μL LDS sample buffer and 10 μL, reducing agent (Invitrogen, Carlsbad, CA, USA) boiled for 10 min and separated on 4–12% Bis–Tris gels (Invitrogen; cat #NP0322). The proteins were transferred onto nitrocellulose membrane (Protean 0.2 μm; Whatman, Dassel Germany), blocked with 5% powdered milk in TBST (10 mm Tris, 150 mm NaCl, 0.15% Tween-20, pH 8.0). Aβ was detected using the 6E10 monoclonal antibody (SIG-39320; Covance Emeryville, CA, USA). ECL was developed using ECL system.

### Early and late life *hsf-1* RNAi efficiency assay

To compare the effects of *hsf-1* RNAi early and late in life worms that express GFP under the regulation of the HSF-1 target gene HSP-16.2 were used (strain CL2070). Synchronized worm populations were developed and grown on EV up to either day 1 or 9 of adulthood and transferred onto *hsf-1* RNAi bacteria for 4 days (*hsf-1* RNAi treatments: either days 1–5 or days 9–13 of adulthood, control worm groups were grown on EV bacteria up to either day 5 or 13 of adulthood). At the last day of treatment, the worms were heat shocked for 6 h at 33 °C (to induce GFP expression), homogenized and cleared by centrifugation (2350 *g*, 5 min, desktop centrifuge). Total protein amounts in the PDS samples were normalized using BCA assay (Pierce, Rockford, IL, USA) and equal amounts were loaded onto 12% PAA gel, transferred onto Polyvinylidene Fluoride (PVDF) membrane and detected using an odyssey imager. Antibodies: affinity purified rabbit anti-GFP was a generous gift from Jill Meisenhelder, α tubulin (Cat # T5168; Sigma, St. Louis, MO, USA). Signal intensities were measured using imagej software.

## Funding

This study was generously supported by the McKnight endowment for neuroscience (AD) and P01 AG031097 (AD and JWK).

## Author contributions

EC and AD designed and initiated this study; EC performed fluorescent detection of DAF-16, paralysis assays, Western blot and quantitative RT-PCR. DD performed *in-vitro* kinetic aggregation assays. EK carried out lifespan experiments and DJ performed a paralysis assay and GFP detection. YV performed quantitative RT-PCR experiments. EC, AD and JWK wrote the manuscript.

## Supporting Information

Additional supporting information may be found in the online version of this article: **Fig. S1** (**A)** Repeat of 1A. **Fig. S2** Statistical data of the lifespan experiment displayed in Figure 1C. **Fig. S3** Aβ worms that were grown on*daf-2* RNAi and transferred onto control bacteria (EV) ateither day 1, 5 or 9 of adulthood were notably protected fromAβ toxicity through day 13 of adulthood, indicating thatremoval of the worms from *daf-2* RNAi does not abolish the counter proteotoxic effect associated with reduced IIS. **Fig. S4** Quantitative RT-PCR analysis of*daf-16* and *hsf-1* expression in L4 wild-type larva(grown at 20°C): (**A)** *daf-16* mRNA levelsare reduced to approximately 50% 3h after transferring the animalsonto *daf-1*6 RNAi bacteria. (**B)** *hsf-1*mRNA levels are reduced by nearly 80% 6 hours after transferringworms onto *hsf-1* RNAi bacteria. **Fig. S5** CL2070 worms were grown and developedon control bacteria and transferred onto *hsf-1* RNAi ateither day 1 or 9 of adulthood. Both worm groups were grown on*hsf-1* RNAi for identical time, four days, before harvesting. GFP contents in the PDS samples of both groups were analyzed using WB. Relative signal intensities were measured using ImageJ software. As a service to our authors and readers, this journal provides supporting information supplied by the authors. Such materials are peer-reviewed and may be re-organized for online delivery, but are not copy-edited or typeset. Technical support issues arising from supporting information (other than missing files) should be addressed to the authors.
