# Supplementary material for: Temporal requirements of insulin/IGF-1 signaling for proteotoxicity protection
Source: Aging Cell. 2010 Apr;9(2):126–34. doi: 10.1111/j.1474-9726.2009.00541.x (PMC3026833; doi:10.1111/j.1474-9726.2009.00541.x)
Supplement: Supplementary file 1 — Additional supporting information may be found in the online version of this article: Fig. S1 (A) Repeat of 1A. Fig. S2 Statistical data of the lifespan experiment displayed in Figure 1C. Fig. S3 Aβ worms that were grown ondaf-2 RNAi and transferred onto control bacteria (EV) ateither day 1, 5 or 9 of adulthood were notably protected fromAβ toxicity through day 13 of adulthood, indicating thatremoval of the worms from daf-2 RNAi does not abolish the counter proteotoxic effect associated with reduced IIS. Fig. S4 Quantitative RT-PCR analysis ofdaf-16 and hsf-1 expression in L4 wild-type larva(grown at 20°C): (A) daf-16 mRNA levelsare reduced to approximately 50% 3h after transferring the animalsonto daf-16 RNAi bacteria. (B) hsf-1mRNA levels are reduced by nearly 80% 6 hours after transferringworms onto hsf-1 RNAi bacteria. Fig. S5 CL2070 worms were grown and developedon control bacteria and transferred onto hsf-1 RNAi ateither day 1 or 9 of adulthood. Both worm groups were grown onhsf-1 RNAi for identical time, four days, before harvesting. GFP contents in the PDS samples of both groups were analyzed using WB. Relative signal intensities were measured using ImageJ software. As a service to our authors and readers, this journal provides supporting information supplied by the authors. Such materials are peer-reviewed and may be re-organized for online delivery, but are not copy-edited or typeset. Technical support issues arising from supporting information (other than missing files) should be addressed to the authors. [file acel0009-0126-SD1.pdf]

Figure S1

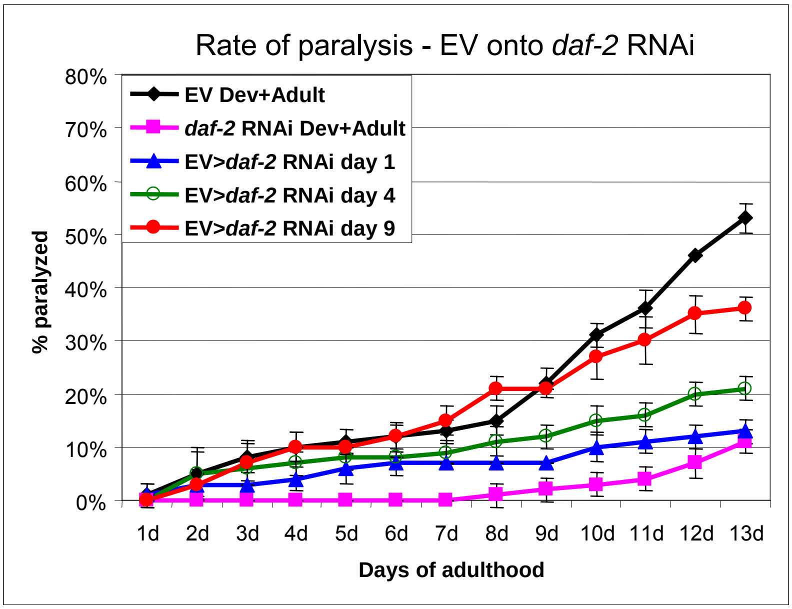

Rate of paralysis - EV onto daf-2 RNAi
80%
70%
60%
50%
40%
30%
20%
10%
0%
% paralyzed
EV Dev+Adult
daf-2 RNAi Dev+Adult
EV>daf-2 RNAi day 1
EV>daf-2 RNAi day 4
EV>daf-2 RNAi day 9
1d
2d
3d
4d
5d
6d
7d
8d
9d
10d
11d
12d
13d
Days of adulthood

Figure S2

EV for life vs *daf-2* RNAi for life

Group	N	N Censored	Mean lifespan (days)	Std Error
EV (life)	81	19	15.481	0.432
daf2 (life)	77	23	23.736	0.737
Combined	158	42	19.523	0.533

Test	ChiSquare	DF	P value
Log-Rank	77.463	1	1.35E-18
Wilcoxon	68.146	1	1.52E-16

EV for life vs *daf-2* RNAi from day 9 of adulthood

Group	N	N Censored	Mean lifespan (days)	Std Error
EV (life)	81	19	15.481	0.432
daf-2(from day9)	86	14	15.353	0.443
Combined	167	33	15.414	0.309

Test	ChiSquare	DF	P value
Log-Rank	3E-05	1	0.99552687
Wilcoxon	0.0836	1	0.7725386

***daf-2* RNAi for life vs *daf-2* RNAi from day 5 of adulthood**

Group	N	N Censored	Mean lifespan (days)	Std Error
daf-2 (from day5)	75	25	17.04	0.612
daf-2 (life)	77	23	23.736	0.737
Combined	152	48	20.452	0.549

Test	ChiSquare	DF	P value
Log-Rank	40.143	1	2.36E-10
Wilcoxon	34.857	1	3.55E-09

Figure S3

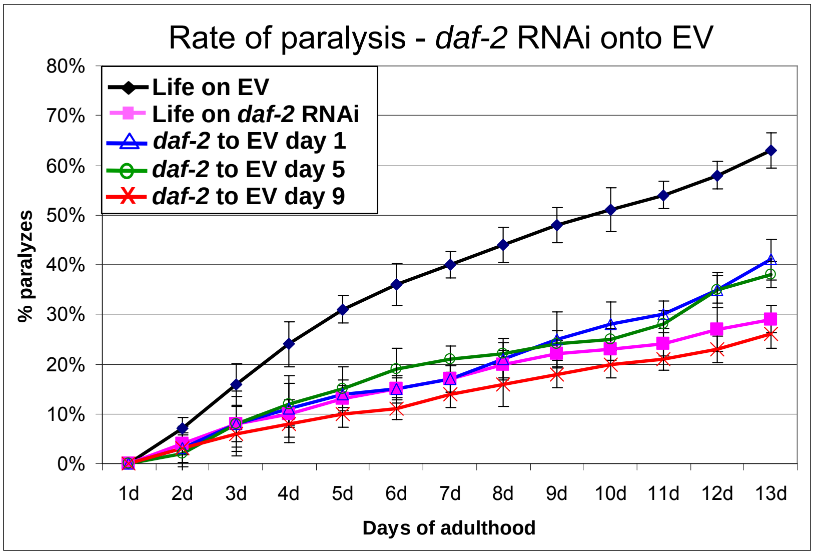

Rate of paralysis - *daf-2* RNAi onto EV
Life on EV
Life on *daf-2* RNAi
daf-2 to EV day 1
daf-2 to EV day 5
daf-2 to EV day 9
% paralyzes
80%
70%
60%
50%
40%
30%
20%
10%
0%
1d
2d
3d
4d
5d
6d
7d
8d
9d
10d
11d
12d
13d
Days of adulthood

Figure S4

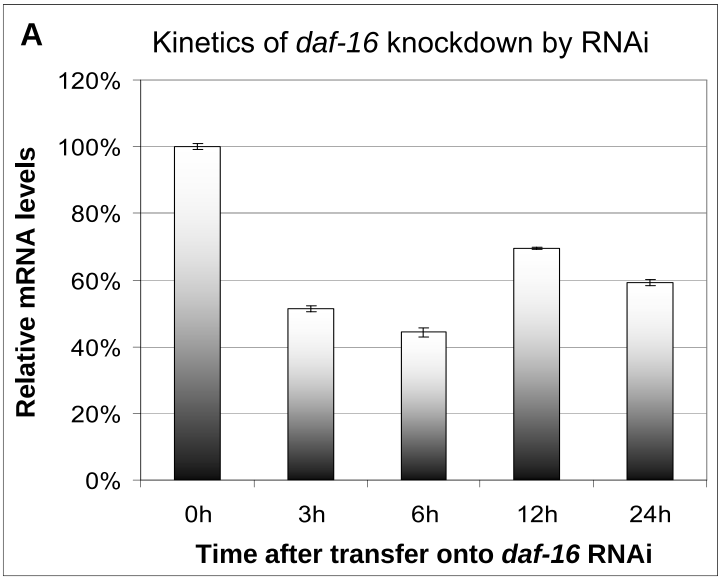
A
Kinetics of daf-16 knockdown by RNAi
120%
100%
80%
60%
40%
20%
0%
Relative mRNA levels
0h
3h
6h
12h
24h
Time after transfer onto daf-16 RNAi

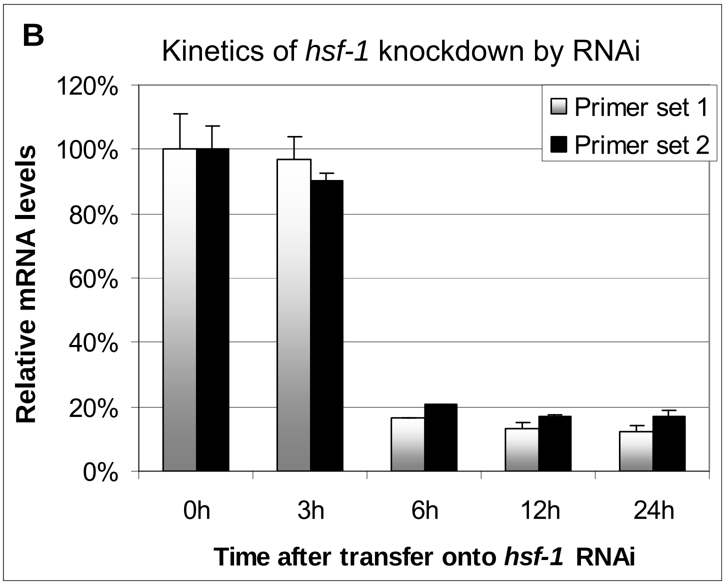
B
Kinetics of hsf-1 knockdown by RNAi
Primer set 1
Primer set 2
120%
100%
80%
60%
40%
20%
0%
Relative mRNA levels
0h
3h
6h
12h
24h
Time after transfer onto hsf-1 RNAi

Figure S5

Age of harvest (day of adulthood)	5 (Young)		13 (old)	
hsf-1 RNAi treatment	—	+	—	+

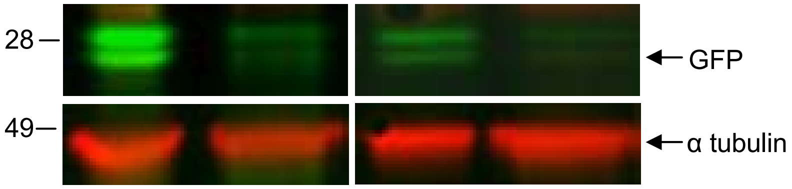

Relative normalized GFP signal intensity	100%	42.4%	100%	47.5%
